# Effect of Coix Seed Extracts on Growth and Metabolism of *Limosilactobacillus reuteri*

**DOI:** 10.3390/foods11020187

**Published:** 2022-01-11

**Authors:** Zhoujie Yang, Anyan Wen, Likang Qin, Yi Zhu

**Affiliations:** 1 Key Laboratory of Plant Resource Conservation and Germplasm Innovation in Mountainous Region (Ministry of Education), Collaborative Innovation Center for Mountain Ecology & Agro-Bioengineering (CICMEAB), College of Life Sciences/Institute of Agro-Bioengineering, Guizhou University, Guiyang 550025, China; zjyangfood_mic@sina.com; 2 School of Liquor and Food Engineering, Guizhou University, Guiyang 550025, China; aywen@gzu.edu.cn; 3 Plant Protection and Plant Quarantine Station of Guizhou Province, Guiyang 550001, China; Zy5286581@126.com

**Keywords:** coix seed extract, *Limosilactobacillus reuteri*, metabolomics, prebiotic

## Abstract

Coix seed (*Coix lachryma-jobi* L.) is an important nourishing food and traditional Chinese medicine. The role of their bioactive constituents in physiology and pharmacology has received considerable scientific attention. However, very little is known about the role of coix seed bioactive components in the growth of *Limosilactobacillus reuteri* (*L. reuteri*). This study aimed to evaluate the effects of coix seed extract (CSE) on the growth, acidifying activity, and metabolism of *L. reuteri*. The results showed that CSE can increase the growth and acidifying activity of *L. reuteri* compared with the control group. During the stationary phase, the viable bacteria in the medium supplemented with coix seed oil (CSO, 13.72 Log_10_ CFU/mL), coix polysaccharide (CPO, 12.24 Log_10_ CFU/mL), and coix protein (CPR, 11.91 Log_10_ CFU/mL) were significantly higher (*p* < 0.05) than the control group (MRS, 9.16 Log_10_ CFU/mL). CSE also enhanced the biosynthesis of lactic acid and acetic acid of *L. reuteri*. Untargeted metabolomics results indicated that the carbohydrate metabolism, amino acid metabolism, and nucleotide metabolism activities of *L. reuteri* were increased after adding CSE. Furthermore, CSE increased the accumulation of bioactive metabolites, such as phenyl lactic acid, vitamins, and biotin. Overall, CSE may have prebiotic potential and can be used to culture *L. reuteri* with high viable bacteria.

## 1. Introduction

Probiotics are live microorganisms that can promote host health [1]. Their role in preventing chronic diseases and regulating intestinal flora has been confirmed [2,3]. At present, probiotics have been used in commercial production mainly including *Lactobacillus*, *Bifidobacterium*, *Lactococus*, *Streptococcus,* and *Enterococcus*. *L. reuteri* is one of the probiotics that has attracted much attention due to its promotion of host health. It has been reported that *L. reuteri* can metabolize glycerol to produce 3-hydroxypropanal with antibacterial activity [4]. In addition, *L. reuteri* can inhibit *Helicobacter pylori*, rebuild the intestinal microbial barrier, and relieve intestinal colic [5,6]. Therefore, *L. reuteri* is usually proposed for the design of functional foods. To date, *L. reuteri* has been used in a variety of foods including dairy products, fermented cereals, and bread [7,8,9].

In probiotic products, the amount of viable bacteria is an important factor to be considered, because the complex gastrointestinal environment is a serious challenge for probiotics. The high cell-density culture of probiotics contributes to increasing their survival rate in the gastrointestinal tract. However, ordinary chemical media can hardly meet the high cell-density cultivation requirements of probiotics. Therefore, various prebiotics had been developed to promote the growth and vitality of probiotics. Fortunately, prebiotics such as oligosaccharides [10] and dietary fibers [11] have been confirmed to promote the growth of probiotics. Among the prebiotics from many sources, plant-based prebiotics are the most widely studied. Kun et al. [12] found that carrot juice significantly promoted the growth and acid production capacity of *bifidobacteria*. Huang et al. [13] reported that polysaccharides from longan pulp promoted the growth of probiotics, and the viable count of bacteria reached 9.12 Log_10_ CFU/mL after 12-h fermentation. Pereira et al. [14] also indicated that *fruta-do-lobo* starch could improve the growth rate of probiotics, and the maximum number of viable bacteria was more than 11 Log_10_ CFU/mL. In addition, some cereal extracts such as rye sprout extracts and wheat extracts promoted probiotic growth and enhanced probiotics acid tolerance [15,16].

Coix seed is the seed of the perennial herbaceous plant coix (*Coix lacryma-jobi* L. var. *mayuen Stapf*) and has attracted wide attention from researchers in food and medicine fields due to its beneficial effects on health. Studies have shown that coix seed is rich in starch [17], proteins [18], free amino acids, dietary fibers [19], vitamins, minerals, phytosterols, and flavonoids [20,21,22] and has a high nutritional value. Clinically, coix seed is also widely applied in arthritis, diarrhea, diuretics, and pain relief [19,23]. In particular, the drug Kanglaite Injection with the main component of coix seed oil had been approved for the complementary treatment of non-small cell lung cancer [24]. Animal experiments showed that coix seed polysaccharide could improve the serum insulin level of diabetic mice and increase the abundance of *Lactobacillus* in mice [25]. Although the beneficial effects of coix seed on health have received extensive scientific attention [26], very few studies have concentrated on the growth-promoting effect of coix seed extract (CSE) for probiotics.

The objectives of the present study were mainly to evaluate the effects of CSE on growth, acidifying activity, and metabolism of *L. reuteri.* We first assessed the effect of adding CSE to MRS medium on the growth and acidifying activity of *L. reuteri*. Next, we investigated the different metabolites of *L. reuteri* in the medium supplemented with CSE using ultra-performance liquid chromatography coupled with time-of-flight mass spectrometry (UPLC-Q-TOF-MS/MS). The results would be helpful to elucidate the promoting effect of CSE on the growth of *L. reuteri* and provide a reference for the development of probiotic products with high viable counts.

## 2. Materials and Methods

### 2.1. Raw Materials and Chemical Reagents

Coix seed was procured from Guizhou Renxin Agriculture Development Co., Ltd. (Guizhou, China). MRS (De Man Rogosa Sharp) broth and MRS agar were purchased from Shanghai Bio-way Technology Co., Ltd. (Shanghai, China). Acetonitrile (ACN) and methanol were of HPLC grade and purchased from Sinopharm Chemical Reagent Co., Ltd. (Suzhou, China). Other chemicals and reagents were of all analytical grade and purchased from Suzhou Sinopharm Chemical Reagent Co., Ltd. (Suzhou, China).

### 2.2. Strains and Culture Conditions

The probiotic organism used in the study was *L. reuteri* BNCC186563 purchased from BeNa Culture Collection (Suzhou, China). The strain was sub-cultured three times for 24 h at 37 °C in MRS agar before the experiment. The starter culture was cultured for 16 h at 37 °C in MRS broth, then centrifuged at 3000 rpm for 5 min, washed with 0.9% saline, and diluted to obtain a preparation with a concentration of about 7 Log_10_ CFU/mL.

### 2.3. Preparation of Coix Seed Extracts

Coix seed oil (CSO) was prepared based on the previous method with minor modifications [27]. Briefly, coix seeds were ground in an electric grinder and sieved with a 60-mesh sieve. Petroleum ether (boiling range 60–90 °C) was added to the coix seed flour at a solid-liquid ratio of 1:8 (*w*/*v*) for 2-h extraction and the extraction solution was refluxed in a water bath of 80 °C. After filtration, the filtrate was collected and then concentrated under reduced pressure to recover petroleum ether at 60 °C. After evaporating the remaining petroleum ether, coix seed oil was obtained.

Coix seed polysaccharides (CPO) were extracted with ultrasound-assisted extraction and the ethanol precipitation method. Distilled water was added to the flour after extracting the CSO (solid-liquid ratio of 1:10 (*w*/*v*)). Ultrasonic extraction was carried out for 50 min under the condition of ultrasonic power of 800 W, followed by reflux extraction at 95 °C for 4 h. After extraction, the filtrate was collected and concentrated. Then, the filtrates were precipitated by adding four times the volume of ethanol (95%) overnight at 4 °C. The precipitates were centrifuged at 6500 rpm for 20 min and washed twice with ethanol and diethyl ether to remove residual fat-soluble components [28].

Coix seed protein (CPR) was extracted by alkali extraction and acid precipitation. Defatted coix seed flour was dispersed in distilled water (solid-liquid ratio of 1:40 (*w*/*v*)). The pH of the mixture was adjusted to 13 with 0.5 mol/L NaOH and stirred intermittently for 3 h at 40 °C. Then, the suspension was centrifuged at 6500 rpm for 15 min at 4 °C. The supernatant was collected and the pH adjusted to 3.5 with 1 mol/L HCl. After the collected supernatant stood for 30 min, precipitated proteins were collected by centrifugation at 6500 rpm for 15 min at 4 °C and washed 3 times with distilled water [29].

### 2.4. Inoculation and Fermentation

Coix seed extracts (CSO, CPR, and CPO) were added into MRS broth according to the ratio of 1% (*w*/*v*). MRS broth without extracts was used as the control group. The prepared substrate was steamed at 121 °C for 20 min and then cooled to room temperature. The substrate was inoculated with 5% (*v*/*v*) of starter cultures with a bacterial count of 7 Log_10_ CFU/mL. All samples were incubated for 24 h under anaerobic conditions.

### 2.5. Determination of pH and Total Reducing Sugar (TRS)

The pH was evaluated every 4 h with a digital pH meter (Testo Instruments (Shenzhen) Co., Ltd., Shenzhen, China). TRS was determined with the dinitrosalicylic acid method [30]. Fermentation samples obtained at different time points were centrifuged at 6000 rpm for 10 min to acquire the supernatant. Dinitrosalicylic acid reagent (700 μL) was added into the supernatant (700 μL), thoroughly mixed, heated in a boiling water bath for 5 min, and cooled to room temperature. The absorbance of samples was measured at 540 nm. The standard curve was established with glucose standard (Beijing Solarbio Science and Technology Co., Ltd., Beijing, China) and TRS in the sample was calculated according to the standard curve. All experiments were performed in triplicate.

### 2.6. Viable Cell Counts

The absorbance of each experimental group at 600 nm (OD_600nm_) was measured with a spectrophotometer to observe the growth of *L. reuteri* every 4 h within 24 h [31]. In the stable period, viable cell counts of *L. reuteri* were obtained by counting the colony-forming units (CFU) on MRS agar [32]. Briefly, the fermented sample (1 mL) was added into 9 mL of sterile saline and serially diluted. The dilution was used for microbial enumeration with MRS agar plates. These plates were cultured anaerobically for 48 h at 37 °C. Results were expressed as Log_10_ CFU/mL. All experiments were performed in triplicate.

### 2.7. Analysis of Organic Acids by HPLC

A high-performance liquid chromatography (HPLC) system equipped with an ultraviolet detector (Agilent, Santa Clara, CA, USA) was used to measure organic acids (lactic acid, acetic acid, L-malic acid, and citric acid). Fermentation samples were centrifuged at 10,000× *g* for 10 min to acquire the supernatants, which were then filtered through a Millex-HA filter with a pore size of 0.22 µm. Mobile phase was 0.02 M KH_2_PO_4_ (pH = 2.7) and the flow rate was 1.0 mL/min. An isocratic elution procedure was adopted. The column temperature was set at 35 °C and the detection wavelength was set as 210 nm [33]. All the organic acids were determined with corresponding pure standards (purchased from Sinopharm Chemical Reagent Co., Ltd. (Suzhou, China)) at different concentrations (lactic acid: 0.25–4.00 mg/mL; acetic acid: 0.25–4.00 mg/mL; L-malic acid: 0.25–4.00 mg/mL; citric acid: 0.25–4.00 mg/mL). The HPLC results were qualitatively analyzed by peak retention time and quantified by peak area using the external standard method. All experiments were performed in triplicate.

### 2.8. Measurement of LDH Activity

The enzyme activity of lactate dehydrogenase (LDH) was measured with a lactate dehydrogenase Kit (Beijing Solarbio Science and Technology Co., Ltd., Beijing, China). Fermentation samples were centrifuged (8000× *g* for 10 min at 4 °C) to collect bacteria. Extracts were added to bacteria, which were disrupted by sonication with an ultrasonic disruptor (Ningbo Scientzbiotechnology Co., Ltd., Ningbo, China). The suspension was centrifuged (8000× *g* for 10 min at 4 °C) to remove cell debris and then the supernatant was collected for enzyme assays. Enzyme activity was measured spectrophotometrically at 450 nm. One unit of enzyme activity (U) of LDH was defined as every 10,000 bacteria releasing 1 nmol of pyruvic acid per minute. All experiments were performed in triplicate.

### 2.9. UPLC-Q-TOF-MS/MS Analysis

The samples (100 μL) were transferred to a 2-mL Eppendorf tube, resuspended in 400 μL of extraction solvent (acetonitrile-methanol, 1:1) by vortexing, and then sonicated for 10 min. The samples were incubated at −20 °C for 1 h and centrifuged at 13,000 rpm and 4 °C for 15 min. The supernatant (350 μL) was transferred to a 1.5-mL Eppendorf tube and dried in a vacuum concentrator. The metabolites were redissolved in the extraction solvent (acetonitrile-water, 1:1), vortexed for 30 s, sonicated for 10 min, and then centrifuged at 13,000 rpm and 4 °C for 15 min. Finally, the supernatant (50 µL) was transferred to LC vials for subsequent liquid chromatography-mass spectrometry (LC-MS) analysis.

Ultra-high-performance liquid chromatography (UPLC) chromatographic separation was performed with a SCIEX UPLC system (Exion LC, SCIEX, Concord, NH, USA) equipped with a Waters UPLC column (ACQUITY UPLC BEH Amide 1.7 µm, 2.1 × 100 mm, Waters, Milford, MA, USA). Mobile phase A was composed of 25 mM ammonium acetate and 25 mM ammonium hydroxide in water and Mobile phase B was composed of 100% ACN. The gradient solution program was set as follows: 95% B, 0.5 min; 95–65% B, 0.5 to 7 min; 65% to 40% B, 7 to 8 min; 40% B, 9 min; 40% to 95% B, 9 to 9.1 min; and 95% B, 12 min. The flow rate was set at 0.5 mL/min. The injection volume was 2 µL and the sample temperature in the autosampler was maintained at 4 °C.

The UPLC system was coupled to a quadrupole-time-of-flight mass spectrometer (QTOF MS; Triple TOF 5600+, SCIEX, Concord, NH, USA) system via an electrospray ionization (ESI) source operated in positive (5500 V) and negative ionization modes (−4500 V). The MS conditions were set as follows: the ion gas temperature at 650 °C, the ion gas pressure at 60 psi, the curtain gas at 30 psi, and the declustering potential at 60 V. TOF MS data were acquired in the *m/z* range of 60 to 1200 at 0.15 s/spectra with collision energy 10 eV and 12 most abundant mass peaks in TOF MS were selected to perform data-dependent acquisition scanning. MS/MS data were recorded over the *m/z* range of 25 to 1200 at 0.03 s/spectra with a collision energy of 30 eV.

### 2.10. Data Processing

To ensure the stability and reliability of metabolomics data, five biological replicates were arranged for each experimental group. The raw data were firstly converted into mzXML formats by ProteoWizard software. Then XCMS was used for retention time correction, peak identification, peak extraction, peak integration, peak alignment, etc. The minfrac was set to 0.5 and the cutoff was set to 0.6. The quantitative results of each sample were used for normalization and the normalized data were analyzed by principal component analysis (PCA) and orthogonal partial least squares discriminant analysis (OPLS-DA) to distinguish variables between groups with calculated variable importance in projection (VIP) value. A VIP value indicates the contribution of each variable to the model. The metabolites with VIP > 1 and *p* < 0.05 (student’s *t*-test) were considered as significantly changed metabolites. In addition, commercial databases including KEGG (http://www.kegg.jp, accessed on 14 August 2021) and MetaboAnalyst (http://www.metaboanalyst.ca/, accessed on 14 August 2021) were utilized to search for the pathways of metabolites.

### 2.11. Statistical Analysis

All the data were expressed as mean values ± standard deviation. Data analyses were conducted with SPSS Version 19.0 software package for Windows. Analysis of variance was conducted through an ANOVA Tukey’s test to determine any significant difference between samples (*p* < 0.05).

## 3. Results and Discussion

### 3.1. Changes in pH and TRS during the Growth of L. reuteri

**Figure 1:**
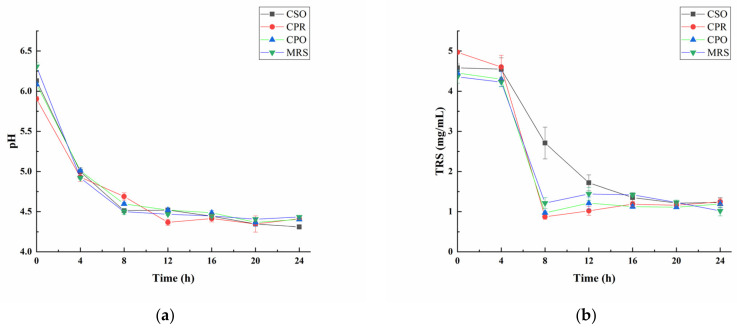
Changes of pH (**a**) and total reducing sugar (TRS) (**b**) during the growth of *L. reuteri*. Values are presented as mean ± standard deviation (*n* = 3).

As shown in Figure 1a, the pH values gradually decreased during the fermentation progresses. At the beginning of fermentation (0 h), the pH value of each group was close to 6.0. At the first 12 h of fermentation, the pH value had dropped significantly. After that, the rate of decline decelerated. To the end of fermentation (24 h), except that the pH in the CSO group was 4.3, the other groups were stabilized at about 4.4. It was reported that the optimal pH for the growth of *L. reuteri* was 4.5 to 6.8 [34], which was close to the pH range in this study. Lactic acid, acetic acid, and CO_2_ produced by the fermentation of *L. reute*ri were the main reasons for the decrease in pH.

Sugar is an important source of energy in the growth and metabolism of microorganisms [35]. Sugar consumption during fermentation can indirectly reflect the growth condition of microorganisms. We analyzed the changes of total reducing sugar (TRS) in four experimental groups (Figure 1b). TRS was significantly reduced after fermentation (*p* < 0.05). In the logarithmic phase, a large amount of TRS was consumed for the rapid proliferation of *L. reuteri*. At the end of the logarithmic phase, the content of TRS increased slightly due to the *L. reuteri-*produced exopolysaccharides [36]. The changes of TRS in the CPO, CPR, and MRS groups were similar. However, the addition of CSO reduced the consumption of reducing the sugar by *L. reuteri*, indicating that CSO might be involved in energy supply, but the mechanism remained to be further explored.

### 3.2. Effect of CSE on the Growth of L. reuteri

**Figure 2:**
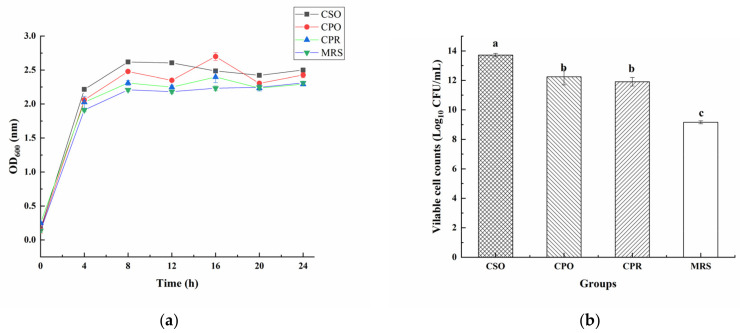
The effect of coix seed extract (CSE) on the growth of *L. reuteri* (**a**) and the count of viable bacteria in the stable phase (**b**). Values are presented as mean ± standard deviation (*n* = 3). Different letters indicate statistical differences between groups (*p* < 0.05).

Figure 2a shows the growth of *L. reuteri* in all substrates. In the first 12 h, *L. reuteri* increased rapidly and then remained at a relatively stable level. Under the same inoculation concentration, the cell density in the medium supplemented with CSE was significantly higher than in the control group (*p* < 0.05). After 24 h of fermentation, the cell density in the group with CSO was the highest. In addition, we analyzed the number of viable cells in the stationary phase (24 h) (Figure 2b). The number of viable bacteria in the CSO group was 13.72 Log_10_ CFU/mL, which was significantly higher than that in CPO (12.24 Log_10_ CFU/mL), CPR (11.91 Log_10_ CFU/mL), and MRS (9.16 Log_10_ CFU/mL). After fermentation, the numbers of viable bacteria in CSO, CPO, and CPR groups were increased by 6 Log_10_ CFU/mL, 5 Log_10_ CFU/mL, and 4 Log_10_ CFU/mL, respectively. The growth-promoting effect of cereal extracts for probiotics has been confirmed [15,37]. Nsogning et al. [38] have shown that wort buffering can promote the growth and viability of probiotics. Chavan et al. [39] confirmed that viable bacteria were more than 11 Log_10_ CFU/mL in probiotic drinks containing cereals. Similar or even better results were observed in our study, indicating that CSE has a good prebiotic effect on *L. reuteri*.

CSO mainly contains oleic acid, linoleic acid, and glycerate. Although some researchers reported that linoleic acid could inhibit the growth of lactic acid bacteria (LAB) [40], many LAB have linoleic acid isomerase, which could convert linoleic acid into conjugated linoleic acid [41]. Other researchers believed that the conversion of free linoleic acid into fatty acid metabolites by LAB might be a detoxification mechanism for enhancing the tolerance to free linoleic acid [42]. Therefore, CSO did not inhibit the proliferation of *L. reuteri*. CPO is mainly composed of arabinose, galactose, mannose, rhamnose, xylose, and glucose [43]. *L. reuteri* has *β*-galactosidase and can utilize lactose as a carbon source to support growth [44]. Zhao et al. [45] also reported that *L. reuteri* could utilize raffinose and sucrose as carbon sources, the utilization of these carbon sources was not inhibited in the presence of glucose. However, *L. reuteri* lacks a complete proteolytic system and has a low utilization rate of proteins [44], so CPR showed a weak effect on the proliferation of *L. reuteri* among the three extracts.

### 3.3. Effects of CSE on Organic Acids

**Figure 3:**
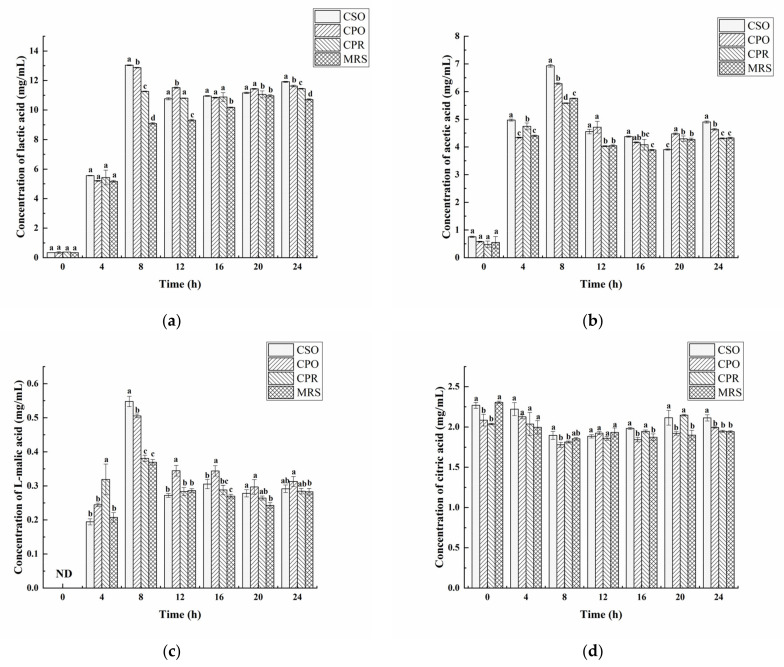
Effects of CSE on organic acids during the growth of *L. reuteri*. Including lactic acid (**a**), acetic acid (**b**), l-malic acid (**c**), and citric acid (**d**). The Values are presented as mean ± standard deviation (*n* = 3). Different letters indicate statistical differences between groups (*p* < 0.05).

Organic acids were determined by HPLC in the fermentation process, including lactic acid, acetic acid, L-malic acid, and citric acid (Figure 3). Compared with MRS, CSE significantly promoted the biosynthesis of lactic acid and acetic acid (*p* < 0.05). The change of lactic acid was shown in Figure 3a, the concentration of lactic acid increased sharply in the early stages (from 4 to 8 h) of fermentation. In the first 4 h, there was no significant difference in lactic acid content among groups (*p* > 0.05). Except for the MRS group, the content of lactic acid reached a maximum in CSO (13.03 mg/mL), CPO (12.87 mg/mL), and CPR (11.26 mg/mL) at 8 h. After that, the lactic acid concentration decreased slightly, and it is possibly involved in the formation of other substances, such as ethyl lactate and ethyl acetate [46]. At the end of fermentation (24 h), the content of lactic acid in CSO was 11.92 mg/mL significantly higher than that in CPO (11.63 mg/mL), CPR (11.45 mg/mL), and MRS (10.72 mg/mL). The analysis of acetic acid showed that CSO and CPO significantly promoted the production of acetic acid in *L. reuteri* (Figure 3b). However, there was no significant difference in acetic acid content between CPR and MRS (*p* > 0.05). During the fermentation process, the changing trend of acetic acid was similar to lactic acid. At 8 h, the content of acetic acid in CSO, CPO, CPR, and MRS were 6.93 mg/mL, 6.29 mg/mL, 5.58 mg/mL, and 5.75 mg/mL, respectively. After 24 h, the content of acetic acid in CSO, CPO, CPR, and MRS were 4.90 mg/mL, 4.64 mg/mL, 4.31 mg/mL, and 4.32 mg/mL, respectively. Salmerón et al. [47] reported the content of lactic acid (2.57 mg/mL) and acetic acid (0.13 mg/mL) fermented with *L. reuteri* in malt beverages, which were significantly lower than our research results. Notably, a higher concentration of lactic acid and acetic acid can inhibit the growth of *L. reuteri.* Under this condition, *L. reuteri* modifies pyruvate metabolism, increasing the synthesis of basic compounds to protect cells against acid stress [48]. Thus, the growth of *L. reuteri* was not inhibited by acid stress.

As shown in Figure 3c, a lower concentration of L-malic acid was detected in the fermented medium. Similar to the change of lactic acid, the content of L-malic acid in each group reached the maximum at 8 h. From 8 to 24 h, the content of L-malic acid was reduced, which might be related to the formation of lactic acid [49]. After 24 h of fermentation, the content of L-malic acid (0.31 mg/mL) was the highest in the medium supplementation with CPO, which was similar to the result reported by Nsogning et al. [38]. In addition, there was no significant difference of L-malic acid in the medium supplementation with CSO, CPR, and MRS (*p* > 0.05).

Figure 3d shows the change of citric acid in the fermentation process. Citric acid detected before fermentation (0 h) might originate from the hydrolysis of citrate. As an important precursor in the tricarboxylic acid cycle, citric acid is eventually metabolized into acetic acid and succinic acid and produces ATP to provide energy for the growth of microorganisms [50]. Therefore, citric acid in each group was decreased in the logarithmic phase. On the contrary, the content of citric acid increased in the later stages of fermentation, indicating that *L. reuteri* might produce citric acid during the fermentation. This finding is in accordance with the Zalán study [51].

To sum up, lactic acid and acetic acid were the main organic acids produced by *L. reuteri.* The acidifying activity of *L. reuter* was increased in the medium supplementation with CSE.

### 3.4. Effect of CSE on LDH Activity during the Growth of L. reuteri

**Figure 4:**
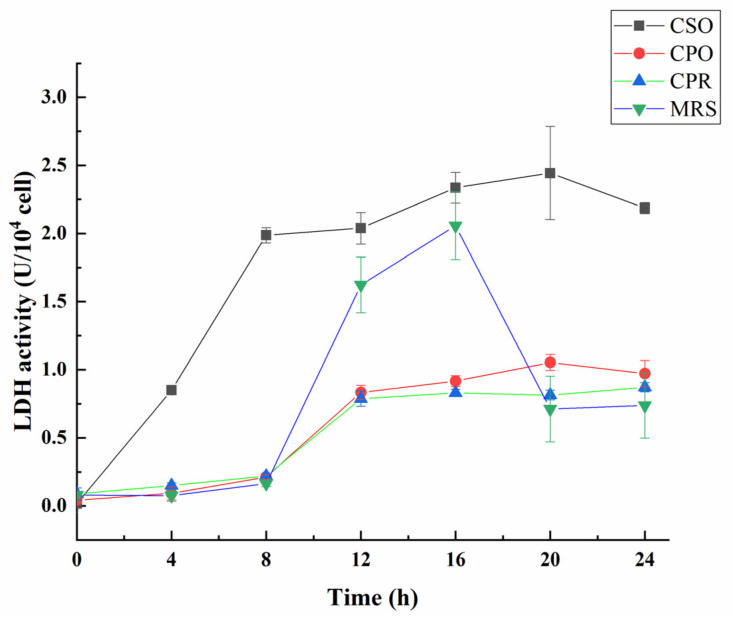
Effect of CSE on lactate dehydrogenase (LDH) activity during the growth of *L. reuteri*. Values are presented as mean ± standard deviation (*n* = 3).

Lactate dehydrogenase (LDH) is a key enzyme in LAB, which can catalyze the reversible reduction of pyruvate to lactic acid [52]. To further clarify the effect of CSE on the acidification activity, LDH activity was measured during the growth of *L. reuteri* (Figure 4). Throughout the fermentation process, the activity of LDH in the CSO group was higher than that in other experimental groups. In the first 8 h of fermentation, the LDH activity of *L. reuteri* rapidly increased in the medium supplemented with CSO, and reached maximum (2.44 U/10^4^ cell) at 20 h. In the CPO and CPR groups, the LDH activity increased at 8 to 12 h. Different from the other groups, the activity of LDH in the MRS increased at 8 to 16 h, and reached a maximum (2.05 U/10^4^ cell) at 16 h. Notably, LDH activity in the MRS was higher than that in the CPO and CPR groups from 12 h to 16 h, which may be related to intracellular pyruvate content [53]. LDH activity of all the experimental groups has a decreasing trend at the late fermentation stage. According to Busto et al. [54], nucleotides (ATP and AMP) competitively occupied the binding sites of pyruvate under the acidic environment, thus inhibiting the activity of LDH. This may be the reason for the decrease of LDH activity in the late fermentation stage. Overall, the acidification activity of *L. reuteri* was significantly increased in the MRS medium Supplemented with CSE.

### 3.5. Results of Principal Component Analysis (PCA) and Orthogonal Partial Least Squares Discriminant Analysis (OPLS-DA) of Cultured Samples from Different Extracts

**Figure 5:**
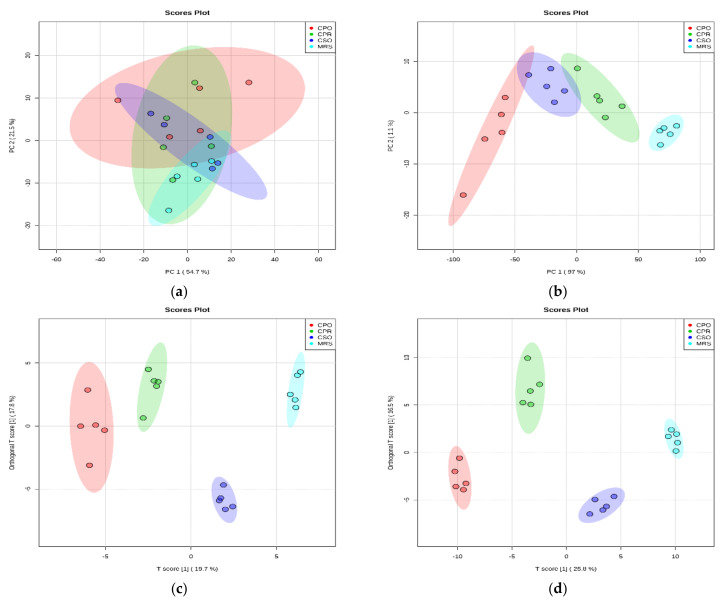
Principal component analysis (PCA) and orthogonal partial least square discriminant analysis (OPLS-DA) of *L. reuteri* metabolites adding CSE in positive mode ion and negative ion mode. (**a**,**b**) are the PCA score plot in positive ion mode and negative ion mode, respectively. (**c**,**d**) are the OPLS-DA score plot in positive ion mode and negative ion mode, respectively.

Unsupervised principal component analysis was performed to explore the different metabolites of *L. reuteri* in the medium supplemented with CSE. The results are shown in Figure 5, the cumulative variance contribution of PC1 and PC2 were 54.7% and 21.5% respectively in positive ion mode (Figure 5a), and the cumulative variance contribution of PC1 and PC2 were 97% and 1.1% respectively in negative ion mode (Figure 5b). The results indicated that there were more similar metabolites between different extracts in the negative ion mode, but there were differences with the control group. In the positive ion mode, there was an obvious discrete phenomenon between groups, without intersection or overlap, indicating that there were significant differences in metabolites among groups. The results of the groups of different extracts and the control group were analyzed (Supplementary Figure S1).

The metabolic differences among the groups of different extracts were further detected by OPLS-DA. In the OPLS-DA score graph (Figure 5c,d), there was a high degree of differentiation among different groups, and metabolic differences existed among the samples. The values of R^2^Y and Q^2^ positive ionization mode were respectively 0.994 and 0.91 and the values of R^2^Y and Q^2^ negative ionization mode were respectively 0.956 and 0.817. The data indicated that the model was reliable and had a good predictive ability. The results of OPLS-DA between the groups of different extracts and the control group were analyzed (Supplementary Figure S2).

### 3.6. KEGG Annotation and Metabolic Pathway Analysis

**Figure 6:**
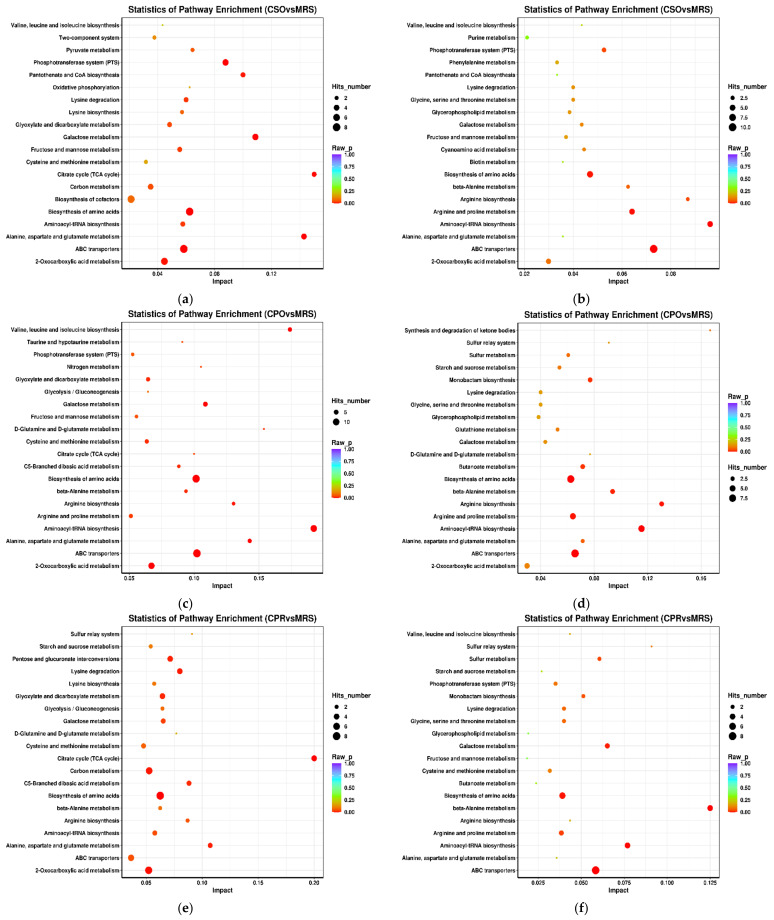
Metabolite enrichment pathway analysis of *L. reuteri* in the medium supplemented with CSE under positive ion mode and negative ion mode. Positive ion mode: (**a**) (CSO vs. MRS), (**c**) (CPO vs. MRS), (**e**) (CPR vs. MRS). Negative ion mode: (**b**) (CSO vs. MRS), (**d**) (CPO vs. MRS), (**f**) (CPR vs. MRS). CSO: coix seed oil; CPO: coix seed polysaccharides; CPR: coix seed protein.

The KEGG database can link metabolites with specific metabolic pathways based on the variation rules of differential metabolites and can be used to explore the metabolic mechanism in vivo and the dynamic changes of organisms [55]. We analyzed the metabolic pathways that were significantly different between the experimental groups and the control group (Figure 6). These metabolic pathways mainly included carbohydrate metabolism, amino acid metabolism, nucleotide metabolism, and membrane transport. Carbohydrate metabolism pathways mainly included galactose metabolism, fructose and mannose metabolism, and starch and sucrose metabolism. Amino acid metabolism pathways mainly include arginine and proline metabolism, alanine, aspartic acid and glutamate metabolism, cysteine and methionine metabolism, valine, leucine, and isoleucine metabolism, and glycine, serine, and threonine metabolism. In addition, related pathways such as aminoacyl-tRNA synthesis, ABC transporter, biotin metabolism, and vitamin metabolism were significantly enriched in experimental groups.

### 3.7. Identification of Different Metabolites

**Figure 7:**
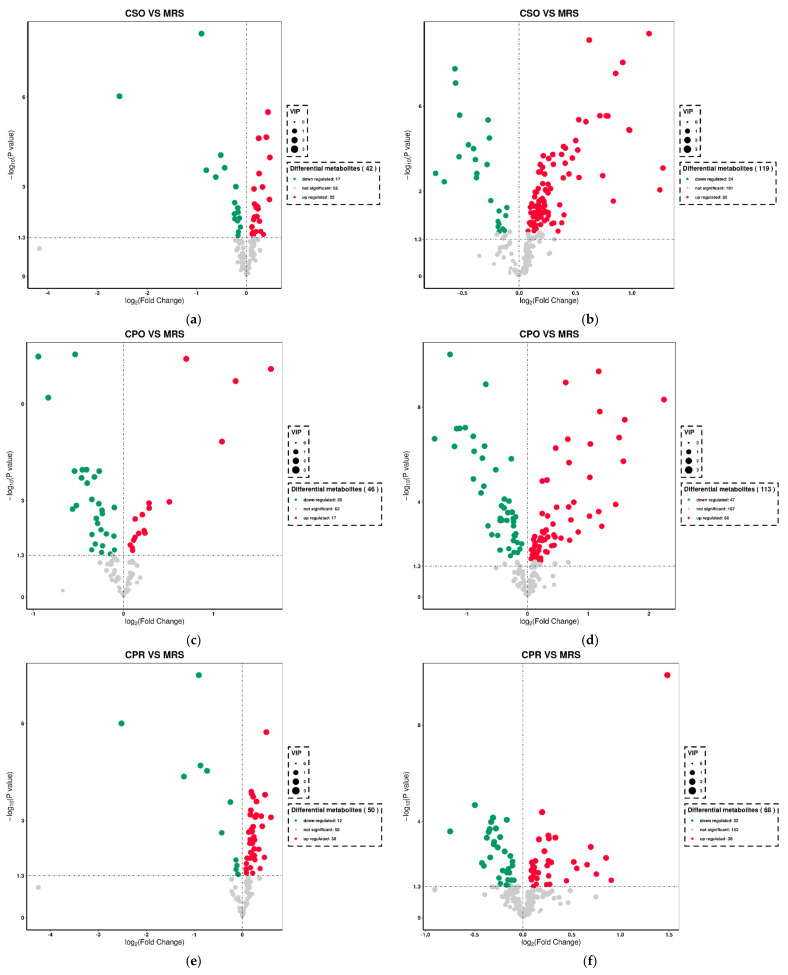
Volcanic plot of differential metabolites of *L. reuteri* in the medium supplemented with CSE under positive ion mode and negative ion mode. Positive ion mode: (**a**) (CSO vs. MRS), (**c**) (CPO vs. MRS), (**e**) (CPR vs. MRS). Negative ion mode: (**b**) (CSO vs. MRS), (**d**) (CPO vs. MRS), (**f**) (CPR vs. MRS).

**Figure 8:**
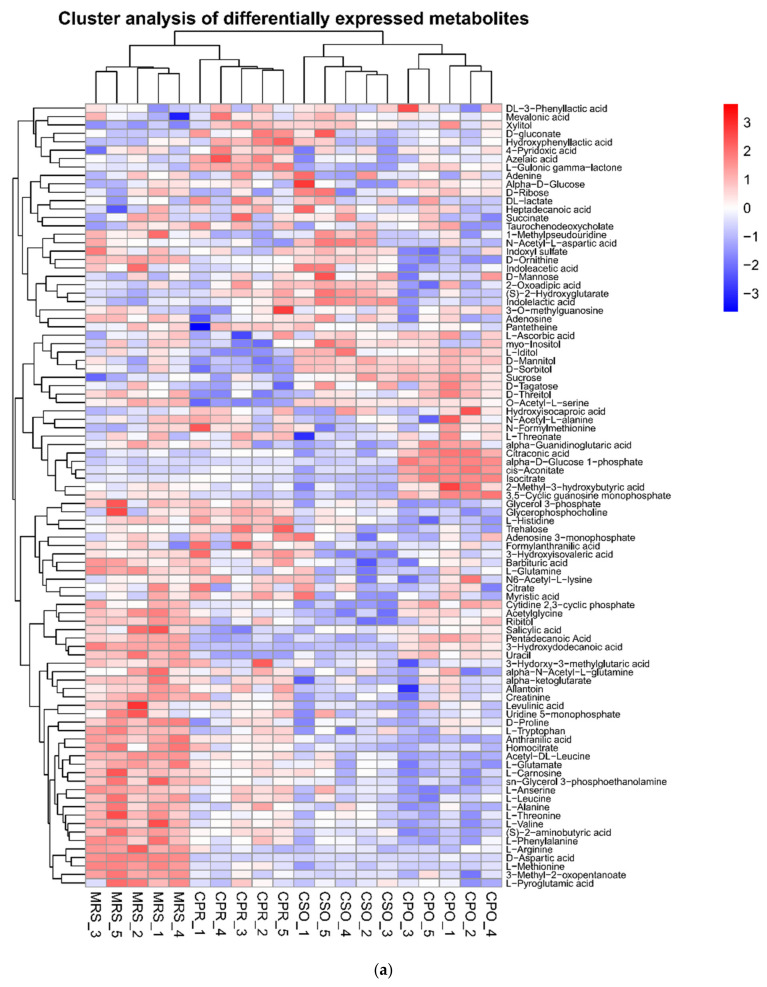

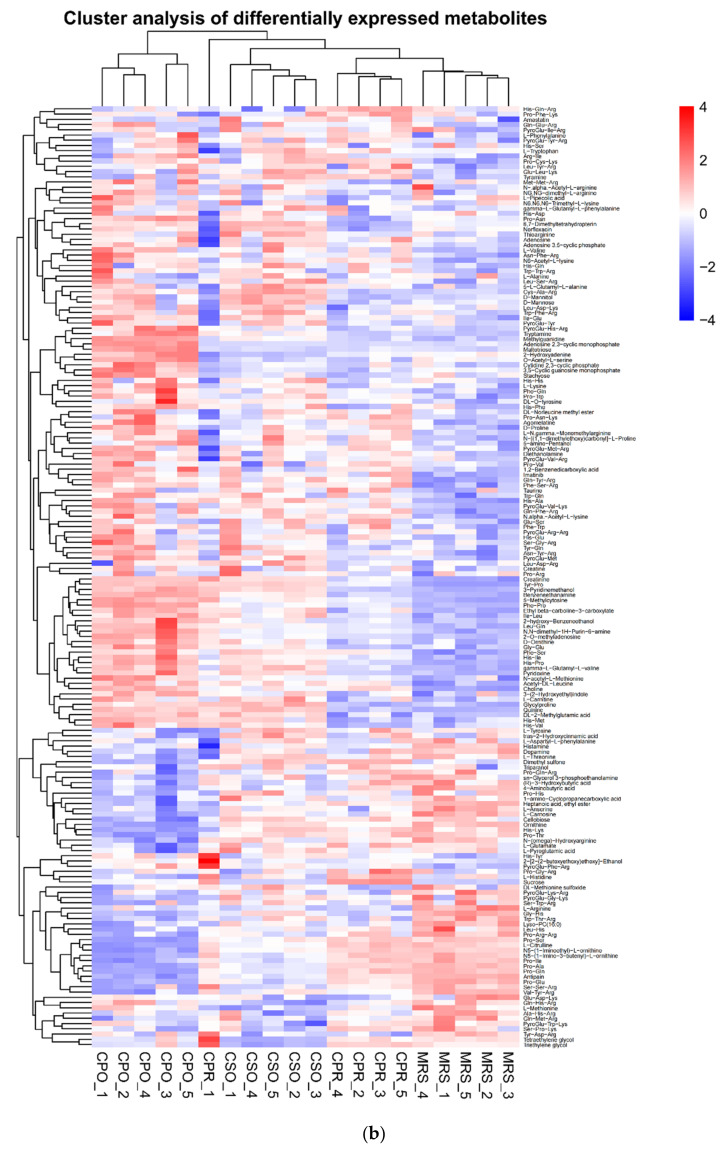
Heatmap of hierarchical clustering analysis of differential metabolites of *L. reuteri* in the medium supplemented with CSE. (**a**) Negative ion mode, (**b**) Positive ion mode.

The metabolites in all experimental groups were identified by UPLC-Q-TOF/MS under positive and negative ionization modes. These metabolites mainly included organic acids, esters, sugars, nucleosides, amino acids, peptides, and alcohols. OPLS-DA was used to analyze the variables of compounds identified by positive and negative ionization modes and the variable importance in projection (VIP) values and *t*-test was combined together to select characteristic metabolites. The metabolites with VIP > 1 and *p* < 0.05 were considered as differential metabolites. Based on the comparison results between experimental groups and the control group, we identified 153, 145, and 109 differential metabolites respectively in CSO (Table S1), CPO (Table S2), and CPR (Table S3) groups. The differential metabolite volcanic map (Figure 7) shows the metabolite changes between the experimental group and the control group. Figure 7a,b shows the changes of different metabolites in the CSO group, including 120 up-regulated metabolites (red dots) and 41 down-regulated metabolites (green dots). In the CPO group, 83 metabolites were up-regulated and 76 metabolites were down-regulated (Figure 7c,d). In the CPR group, 74 metabolites were up-regulated and 44 metabolites were down-regulated (Figure 7e,f). Hierarchical clustering analysis between different experimental groups and control groups was performed to explore the changes of the same metabolites (Figure 8). Our results showed that the contents of some metabolites such as organic acids, alcohols, vitamins, and nucleotides in experimental groups were significantly higher than those in the control group (*p* < 0.05). The contents of amino acids, polypeptides, and pyrimidines were significantly reduced (*p* < 0.05). Cell proliferation required a lot of amino acids, peptides, and nucleosides during the proliferation process of *L. reuteri*. Notably, the abundance of the same metabolite varied significantly among different experimental groups, indicating that the extracts of coix seed led to different proliferative activities of *L. reuteri.*

#### 3.7.1. Carbohydrate Metabolism

Carbohydrates are an important source of energy for the growth of LAB. LAB can metabolize carbohydrates into lactic acid, acetic acid, carbon dioxide, ethanol, etc. Carbohydrate transport is mediated by the members of the ATP-binding cassette (ABC) superfamily of ABC-transporters, secondary transporters of the Major Facilitator Superfamily (MFS), or phospho-transferase systems (PTS) in bacteria [45]. Therefore, ABC transporters and phosphotransferase system-related metabolic pathways were significantly upregulated in experimental groups (Figure 6). Monosaccharides and oligosaccharides are preferentially used in the fermentation process of LAB. In this study, galactose metabolism, sucrose metabolism, and starch metabolism were significantly enriched in carbohydrate-related metabolic pathways. Tagatose, sucrose, and D-glucose were significantly upregulated in CSO and CPO groups. It was reported that L-arabinose isomerase (L-AI) and D-xylose isomerase (D-XI) were found in *L. reuteri*. L-AI is also known as D-galactose isomerase and can convert D-galactose to D-tagatose [56]. Tagatose was catalyzed by tagatose-6-phosphokinase and tagatose-1, 6-diphosphate aldolase to generate glyceraldehyde 3-phosphate, which is involved in the glycolysis pathway [57]. Leite et al. [58] showed that tagatose can promote the growth of Bifidobacterium infantis NRRL B-41661. Therefore, tagatose may also promote the growth of *L. reuteri*. D-XI isomerized D-glucose to D-fructose, which reacted with glucose to form sucrose under the action of enzymes, thus leading to the upregulation of sucrose. Mannose was significantly upregulated in the CSO and CPR groups. Wongsiridetchai et al. [59] found that mannose could promote the growth of LAB and enhance the survival rate of LAB under gastrointestinal conditions. Here, mannose may promote the growth of *L. reuteri*. In conclusion, compared with CPO and CPR, more fermentable sugars were detected in medium supplemented with CSO, which might be one of the reasons for the higher viable count in CSO.

#### 3.7.2. Amino Acid and Peptide Metabolism

Amino acids play an important role in the growth of LAB [60]. After 24 h of fermentation, the contents of some amino acids decreased significantly (Figure 8), including methionine, arginine, valine, threonine, citrulline, isoleucine, and aspartic acid. Safari et al. [61] reported that LAB consumed a lot of alanine, arginine, leucine, and isoleucine for growth, and similar results were observed in this study. Other researchers reported that leucine and isoleucine could increase the biomass of *Lactobacillus* and promote the growth of probiotics [62]. Among these amino acids, arginine produced ornithine through the arginine deiminase pathway (ADI) and released ATP to provide cell energy [63]. Rollan et al. [64] found that *L. reuteri* could metabolize arginine through the ADI pathway. The ADI system is highly resistant to acids, thus enabling *L. reuteri* to grow in an acidic environment. As a sulfur-containing essential amino acid, methionine plays an indispensable role in protein synthesis, modification, and catalytic regulation [65]. In addition, methionine has an antioxidant capacity [66] and can enhance the tolerance of *L. reuteri* to oxygen. N-formylmethionine is an important derivative of methionine in the initial stage of protein synthesis and was significantly upregulated in experimental groups with extracts. Aspartic acid is the precursor of some essential amino acids such as lysine, threonine, methionine, and isoleucine [67]. Aspartic acid can provide essential amino acids for cell growth when essential amino acids for cell growth are limited. Aspartic acid is also involved in the synthesis of purines and pyrimidines [68]. In addition to these down-regulated amino acids, phenylalanine was significantly up-regulated in experimental groups. During the fermentation process, phenylalanine shares a metabolic pathway with lactic acid. Under the action of lactate dehydrogenase, phenylalanine is metabolized into phenyl lactic acid [69]. Phenyl lactic acid has a strong antifungal activity [70]. The metabolism of phenylalanine by *L. reuteri* produces phenyl lactic acid [71]. Gánzle pointed out that the intracellular amino acid level and amination were important factors affecting the accumulation of phenyl lactic acid, which was stimulated by adding peptides, citric acid, and α-ketoglutaric acid [72]. In our study, the contents of phenylalanine, polypeptide, and citric acid in experimental groups were significantly higher than those in the control group, indicating that the content of phenyl lactic acid accumulated in the experimental groups was higher than that in the control group. Besides amino acids, some peptides mainly including short peptides were also involved in the growth and metabolism of *L. reuteri.* These peptides were hydrolyzed to produce amino acids associated with the growth of *L. reuteri*, such as arginine, threonine, and alanine. According to our results, *L. reuteri* prefers utilizing free amino acids as the carbon source for growth, but it seldom utilizes peptides, especially large polypeptides. This might be related to the lack of enzymes to hydrolyze larger peptides in some LAB [73].

#### 3.7.3. Nucleotide Metabolism

Nucleotides are substrates for RNA and DNA synthesis and the material basis for cell division. *L. reuteri* also requires a large amount of nucleotides to maintain cell proliferation. After 24 h of fermentation, the content of uracil in the experimental groups was lower than that in the control group. Pyrimidine is one of the abundant metabolites in cells and plays an important role in cell energy production and cell signal transduction [74]. Many LAB are auxotrophic to purines and pyrimidines and cannot reduce ribonucleotides to the corresponding deoxyribonucleotide for DNA synthesis. However, they possess a special salvage system based on a *trans-N*-deoxyribosylase and the system requires deoxynucleoside in combination with pyrimidine and purine bases [75]. The increased demand for uracil indicated that coix seed extracts might enhance the proliferation ability of *L. reuteri.* In addition, 3,5–cyclic guanosine monophosphate (cGMP), a cellular second messenger signaling molecule, was decreased in the CSO and CPR groups. Another second messenger signaling molecule, adenosine 3,5–cyclic phosphate (cAMP), was increased in the CPO group. cGMP and cAMP are second messenger signaling molecules widely presented in bacteria, especially in biofilms [76]. Under anaerobic conditions, intracellular guanosine had a higher concentration and was easily absorbed [77], thus, cGMP was decreased in experimental groups. Adenosine and guanosine could also significantly stimulate probiotic growth under anaerobic conditions [78]. In addition, the contents of 5-methylcytosine, 2-O-methyladenosine, adenine nucleotides, and other substances in the experimental groups were also higher than those in the control group.

#### 3.7.4. Other Metabolism Pathways

In addition to carbohydrates, nucleotides, and amino acids for growth, other growth factors of *L.reuteri* are also required, such as vitamins and biotin. Vitamins are involved in many functions of the body, including cell metabolism and nucleic acid synthesis [79]. We observed that the content of vitamin B6 (VB6) in the experimental groups was significantly higher than that in the control group (Figure 8). It is reported that VB6 can promote the growth of LAB [80]. The up-regulation of VB6 might contribute to the proliferation of *L.reuteri.* In addition, another vitamin complex (choline) was also significantly upregulated. Baliarda et al. [81] found that choline could alleviate the inhibition of the salt stress on the growth of LAB because choline is a positively charged compound and can effectively balance counter ions in cells and play a protective role under salt stress [82]. In addition, choline is also a component of lecithin, which is of great significance to the formation of cell membranes. The lack of choline can cause cell apoptosis. The biomass of *L. reuteri* in experimental groups was higher than that in the control group probably due to the presence of choline. Notably, inositol was accumulated in the CSO group. Inositol is a precursor to phosphatidylinositol and can enhance cell tolerance to the environment, especially the ethanol stress [83]. Ethanol is one of the by-products in *L. reuteri* fermentation. The presence of inositol might enhance the tolerance of *L. reuteri* to ethanol. Interestingly, besides the normal metabolites of LAB, imatinib was also detected after fermentation. Imatinib is widely used in the treatment of chronic myeloid leukemia [84]. However, whether imatinib is produced by *L. reuteri* fermentation needs further experimental confirmation.

## 4. Conclusions

This study demonstrated that CSE could promote acid production and the growth of *L. reuteri*. Meanwhile, CSE also significantly affected several metabolic pathways of *L. reuteri*, including carbohydrate metabolism, amino acid metabolism, nucleotide metabolism, and vitamin metabolism. These findings suggest that CSE may have prebiotic potential and can be used to culture *L. reuteri* with high viable bacteria. However, further studies are needed using CSE as a single carbon or nitrogen source to determine the growth-promoting effect of CSE on *L. reuteri*. Additionally, more research is needed to confirm the prebiotic effects of CSE on other probiotics. If its prebiotic activity is proven, CSE could be used as a functional food, giving products a healthy appeal.

## Data Availability

Data is contained within the article.

## Supplementary Materials

The following supporting information can be downloaded at: https://www.mdpi.com/article/10.3390/foods11020187/s1, Table S1: The differential metabolites of *L. reuteri* in CSO were determined by UPLC-Q-TOF-MS/MS, based on VIP > 1, *p* < 0.05. Table S2: The differential metabolites of *L. reuteri* in CPO were determined by UPLC-Q-TOF-MS/MS, based on VIP > 1, *p* < 0.05. Table S3: The differential metabolites of *L. reuteri* in CPR were determined by UPLC-Q-TOF-MS/MS, based on VIP > 1, *p* < 0.05. Figure S1: Principal component analysis of differential metabolites in different extracts of *L. reuteri* under positive ion mode and negative ion mode. Positive ion mode: A (CSO vs. MRS), C (CPO vs. MRS), E (CPR vs. MRS). Negative ion mode: B (CSO vs. MRS), D (CPO vs. MRS), F (CPR vs. MRS). Figure S2: Orthogonal partial least square discriminant analysis of differential metabolites in different extracts of *L. reuteri* under positive ion mode and negative ion mode. Positive ion mode: A (CSO vs. MRS), C (CPO vs. MRS), E (CPR vs. MRS). Negative ion mode: B (CSO vs. MRS), D (CPO vs. MRS), F (CPR vs. MRS).
