# Supplementary material for: Effect of Coix Seed Extracts on Growth and Metabolism of Limosilactobacillus reuteri
Source: Foods. 2022 Jan 11;11(2):187. doi: 10.3390/foods11020187 (PMC8774368; doi:10.3390/foods11020187)
Supplement: Supplementary file 1 [file foods-11-00187-s001.zip › foods-1531019-supplementary.pdf]

foods MDPI

Article

Effect of Coix Seed Extracts on Growth and Metabolism of *Limosilactobacillus reuteri*

Zhoujie Yang [1], Anyan Wen [2], Likang Qin [2,*] and Yi Zhu [3]

[1] Key Laboratory of Plant Resource Conservation and Germplasm Innovation in Mountainous Region (Ministry of Education), Collaborative Innovation Center for Mountain Ecology & Agro-Bioengineering (CICMEAB), College of Life Sciences/Institute of Agro-Bioengineering, Guizhou University, Guiyang 550025, China; zsjyangfood_mic@sina.com
[2] School of Liquor and Food Engineering, Guizhou University, Guiyang 550025, China; aywen@gzu.edu.cn
[3] Plant Protection and Plant Quarantine Station of Guizhou Province, Guiyang 550001, China; Zy5286581@126.com
* Correspondence: lkqin@gzu.edu.cn; Tel./Fax: +86-0851-83856374

Table S1. The differential metabolites of *L. reuteri* in CSO were determined by UPLC-Q-TOF-MS/MS, based on VIP > 1, *P* < 0.05.

Compound	mzmed	rtmed	VIP*	P_VALUE	FOLD_CHANGE	Ion mode**
3-Hydroxydodecanoic acid	197.16	66.65	1.62	9.33E-07	0.17	N
D-Aspartic acid	132.03	451.59	1.65	7.53E-09	0.54	N
Uracil	111.02	138.31	1.50	2.79E-04	0.57	N
Pentadecanoic Acid	241.22	63.18	1.47	4.74E-04	0.65	N
L-Arginine	173.10	425.24	1.55	8.71E-05	0.70	N
Acetylglycine	116.04	354.37	1.49	2.30E-04	0.74	N
Cytidine 2,3-cyclic phosphate	304.04	378.71	1.28	8.03E-03	0.85	N
Acetyl-DL-Leucine	172.10	250.00	1.37	3.39E-03	0.85	N
Anthranilic acid	136.04	142.46	1.26	1.19E-02	0.85	N
L-Methionine	148.05	374.86	1.47	9.86E-04	0.86	N
alpha-Guanidinoglutaric acid	188.06	413.37	1.23	1.37E-02	0.89	N
3-Methyl-2-oxopentanoate	129.04	357.76	1.37	5.12E-03	0.89	N
Homocitrate	187.03	414.64	1.11	4.25E-02	0.89	N
3,5-Cyclic guanosine monophosphate	344.05	375.41	1.30	7.06E-03	0.89	N
(S)-2-aminobutyric acid	102.06	442.68	1.16	3.23E-02	0.89	N
Isocitrate	173.01	411.68	1.30	1.12E-02	0.91	N
L-Valine	116.07	367.92	1.15	2.21E-02	0.92	N
Citrate	191.02	643.62	1.16	2.26E-02	1.08	N
1-Methylpseudouridine	272.09	198.57	1.18	2.17E-02	1.08	N
Pantetheine	277.13	75.07	1.08	3.77E-02	1.08	N
3-O-methylguanosine	296.11	263.28	1.05	3.41E-02	1.09	N
Hydroxyphenyllactic acid	181.06	256.68	1.07	4.29E-02	1.11	N
DL-3-Phenyllactic acid	165.06	120.87	1.20	1.25E-02	1.11	N
L-Ascorbic acid	175.03	224.85	1.41	1.18E-03	1.11	N
Succinate	117.02	424.31	1.27	1.02E-02	1.12	N
Hydroxyisocaproic acid	131.07	151.72	1.32	3.77E-03	1.12	N
D-gluconate	195.06	400.12	1.11	3.17E-02	1.15	N
D-Tagatose	179.06	291.67	1.32	4.89E-03	1.15	N
DL-lactate	89.03	237.36	1.34	4.23E-03	1.15	N
N-Acetyl-L-aspartic acid	174.05	433.09	1.25	9.83E-03	1.16	N
L-Iditol	163.06	362.67	1.32	4.74E-03	1.17	N
2-Oxoadipic acid	141.02	549.18	1.56	2.37E-05	1.19	N

D-Sorbitol	182.08	365.29	1.48	3.61E-04	1.20	N
Heptadecanoic acid	269.26	660.71	1.26	1.41E-02	1.20	N
myo-Inositol	161.05	228.75	1.11	3.11E-02	1.22	N
D-Mannitol	181.08	405.42	1.44	1.01E-03	1.25	N
Mevalonic acid	147.07	293.49	1.07	3.91E-02	1.27	N
Indolelactic acid	204.07	197.72	1.56	2.19E-05	1.32	N
Xylitol	151.06	304.85	1.60	3.17E-06	1.35	N
Sucrose	341.12	275.96	1.38	2.66E-03	1.38	N
(S)-2-Hydroxyglutarate	147.03	440.62	1.52	1.04E-04	1.39	N
Pro-Glu	245.11	465.00	1.33	2.33E-04	0.60	P
Tetraethylene glycol	195.11	76.74	1.43	4.75E-08	0.67	P
Pro-Ala	187.11	406.38	1.43	1.52E-07	0.68	P
N5-(1-Imino-3-butenyl)-L-ornithine	263.14	296.80	1.36	6.02E-05	0.69	P
Triethylene glycol	151.10	84.93	1.42	2.06E-06	0.69	P
Antipain	604.35	189.79	1.38	2.30E-05	0.73	P
"Heptanoic acid, ethyl ester"	355.22	168.03	1.37	3.12E-05	0.76	P
2-Hydroxyadenine	152.06	301.87	1.36	7.62E-05	0.76	P
Ser-Ser-Arg	349.18	108.52	1.31	3.32E-04	0.77	P
Pro-Ile	229.15	335.86	1.32	2.35E-04	0.77	P
L-Citrulline	217.12	395.52	1.35	1.13E-04	0.82	P
Pro-Gln	244.13	413.11	1.41	3.06E-06	0.83	P
N5-(1-Iminoethyl)-L-ornithine	215.14	353.45	1.39	1.32E-05	0.83	P
Val-Tyr-Arg	437.23	138.22	1.22	2.14E-03	0.84	P
Pro-Ser	203.10	421.97	1.05	1.78E-02	0.88	P
Pro-Arg-Arg	428.25	448.49	1.09	1.15E-02	0.88	P
Gln-His-Arg	440.21	467.42	1.08	1.45E-02	0.88	P
L-Carnosine	227.11	491.80	1.17	4.96E-03	0.88	P
Lyso-PC(16:0)	496.34	250.10	1.00	2.60E-02	0.89	P
Tyr-Asp-Arg	453.21	130.45	1.04	2.12E-02	0.91	P
Histamine	112.09	633.22	1.02	2.43E-02	0.91	P
4-Aminobutyric acid	104.07	443.02	1.18	3.76E-03	0.93	P
Diethanolamine	70.06	380.52	1.02	2.51E-02	1.06	P
5-amino-Pentanol	86.10	338.99	1.20	3.65E-03	1.07	P
Pro-Asn-Lys	358.20	414.87	1.16	5.67E-03	1.07	P
Agomelatine	244.14	557.20	1.06	1.53E-02	1.07	P
PyroGlu-Arg-Arg	442.22	504.03	1.07	1.28E-02	1.08	P
D-Proline	116.07	404.43	1.09	1.25E-02	1.08	P
PyroGlu-Val-Arg	385.21	422.67	1.07	1.56E-02	1.08	P
DL-Norleucine methyl ester	184.07	542.63	1.22	2.73E-03	1.08	P
tras-2-Hydroxycinnamic acid	165.05	411.19	1.08	1.36E-02	1.09	P
L-Phenylalanine	166.09	335.80	1.14	7.99E-03	1.09	P
Creatine	132.07	439.73	1.08	1.26E-02	1.09	P
Leu-Asp-Lys	375.21	476.37	1.02	2.50E-02	1.10	P
His-Tyr	301.13	252.22	1.03	2.17E-02	1.10	P
Adenosine	268.10	326.43	1.18	3.37E-03	1.10	P
Pro-Asn	230.11	389.82	1.06	1.71E-02	1.10	P
PyroGlu-Tyr	293.11	308.97	1.17	5.60E-03	1.10	P
Glu-Leu-Lys	371.23	410.67	1.25	1.58E-03	1.10	P
Asn-Phe-Arg	436.21	384.40	1.26	1.06E-03	1.10	P
His-Phe	285.13	205.62	1.07	1.55E-02	1.11	P
PyroGlu-Met-Arg	417.18	448.60	1.14	6.39E-03	1.11	P
Gln-Glu-Arg	432.21	503.23	1.03	2.08E-02	1.11	P

L-N.gamma.-Monomethylarginine	227.10	382.45	1.27	1.08E-03	1.11	P
His-His	275.12	360.98	1.18	3.65E-03	1.12	P
L-Lysine	129.10	313.46	1.10	9.55E-03	1.12	P
Trp-Phe-Arg	508.24	360.32	1.16	5.22E-03	1.12	P
Imatinib	494.26	338.38	1.27	8.02E-04	1.12	P
His-Gln	266.12	340.24	1.20	2.91E-03	1.13	P
Adenosine 3,5-cyclic phosphate	362.08	342.84	1.16	5.18E-03	1.13	P
Gln-Phe-Arg	450.23	369.11	1.33	2.09E-04	1.13	P
Asn-Tyr-Arg	452.21	386.12	1.22	2.24E-03	1.13	P
N6-Acetyl-L-lysine	189.12	415.67	1.18	4.21E-03	1.13	P
Tyramine	120.08	325.26	1.35	1.14E-04	1.14	P
Leu-Asp-Arg	403.23	447.98	1.07	1.51E-02	1.14	P
PyroGlu-Val-Lys	357.21	418.06	1.33	2.07E-04	1.14	P
N-[(1,1-dimethylethoxy)carbonyl]-L-Proline	279.13	339.25	1.23	1.99E-03	1.14	P
gamma-L-Glutamyl-L-phenylalanine	277.12	275.99	1.06	1.51E-02	1.14	P
His-Val	237.12	246.73	1.12	8.13E-03	1.14	P
His-Glu	267.11	384.61	1.22	1.79E-03	1.15	P
Norfloxacin	302.13	430.90	1.34	1.42E-04	1.15	P
Gln-Tyr-Arg	466.20	397.47	1.29	5.39E-04	1.16	P
Thioarginine	209.09	326.32	1.15	5.42E-03	1.16	P
PyroGlu-His-Arg	423.20	300.60	1.20	3.54E-03	1.16	P
Choline	104.11	359.99	1.36	5.59E-05	1.16	P
Pro-Trp	302.15	309.50	1.15	5.70E-03	1.16	P
D-Ornithine	115.09	307.28	1.22	2.22E-03	1.16	P
Phe-Ser-Arg	409.21	357.90	1.21	2.50E-03	1.17	P
Arg-Ile	326.17	328.47	1.10	1.11E-02	1.17	P
N.alpha.-Acetyl-L-lysine	230.15	524.41	1.33	2.47E-04	1.17	P
5-L-Glutamyl-L-alanine	201.09	365.55	1.21	3.19E-03	1.17	P
2-hydroxy-Benzeneethanol	121.06	299.04	1.16	5.06E-03	1.17	P
L-Carnitine	162.11	424.40	1.27	8.81E-04	1.18	P
Glu-Ser	217.08	376.65	1.26	9.28E-04	1.18	P
Tyr-Gln	292.13	247.05	1.10	9.60E-03	1.19	P
Dimethyl sulfone	226.99	479.66	1.27	9.75E-04	1.19	P
Ile-Glu	243.13	282.67	1.26	1.15E-03	1.19	P
PyroGlu-Phe-Arg	433.20	106.36	1.28	5.66E-04	1.19	P
Pro-Cys-Lys	346.18	316.80	1.14	7.19E-03	1.20	P
Leu-Ser-Arg	375.22	549.61	1.15	5.61E-03	1.20	P
His-Ile	251.15	205.34	1.36	6.99E-05	1.20	P
PyroGlu-Met	261.09	313.49	1.27	8.08E-04	1.21	P
His-Met	269.10	201.39	1.07	1.40E-02	1.22	P
6,7-Dimethyltetrahydropterin	242.11	385.20	1.36	5.02E-05	1.23	P
D-Mannose	198.09	371.05	1.34	1.12E-04	1.23	P
Phe-Gln	276.13	190.01	1.07	1.31E-02	1.24	P
N-acetyl-L-Methionine	192.07	249.48	1.01	2.55E-02	1.27	P
DL-O-tyrosine	245.09	148.35	1.21	3.01E-03	1.28	P
DL-2-Methylglutamic acid	144.07	250.34	1.36	5.01E-05	1.30	P
Phe-Trp	334.14	294.58	1.08	1.30E-02	1.30	P
Methylguanidine	74.06	159.77	1.30	3.37E-04	1.31	P
His-Ala	209.10	270.46	1.37	2.63E-05	1.31	P
Gly-Glu	204.09	149.58	1.15	6.83E-03	1.32	P
Leu-Gln	242.15	166.90	1.34	1.02E-04	1.33	P
2-O-methyladenosine	282.12	166.06	1.38	2.98E-05	1.33	P

Cys-Ala-Arg	349.16	589.54	1.39	1.62E-05	1.42	P
Phe-Ser	235.11	120.00	1.37	3.50E-05	1.43	P
gamma-L-Glutamyl-L-valine	229.12	328.05	1.41	2.98E-06	1.44	P
N,N-dimethyl-1H-Purin-6-amine	164.09	93.94	1.31	3.27E-04	1.44	P
His-Pro	235.12	194.95	1.40	3.49E-06	1.51	P
Creatinine	114.06	260.49	1.44	4.60E-09	1.54	P
5-Methylcytosine	126.07	251.86	1.41	2.16E-06	1.64	P
Tryptamine	224.12	243.75	1.31	2.82E-04	1.67	P
Quinine	325.19	255.05	1.41	2.17E-06	1.71	P
Pyridoxine	170.08	127.54	1.41	2.19E-06	1.73	P
Leu-Tyr-Arg	451.25	295.69	1.23	2.22E-03	1.78	P
Benzeneethanamine	185.11	242.33	1.43	6.93E-08	1.81	P
3-Pyridinemethanol	110.06	87.17	1.43	2.83E-08	1.89	P
Glycylproline	214.12	241.69	1.40	6.77E-06	1.96	P
Phe-Pro	245.13	67.59	1.39	7.06E-06	1.97	P
Tyr-Pro	261.12	132.27	1.44	2.73E-09	2.22	P
Ile-Leu	227.17	62.59	1.27	8.93E-04	2.37	P
Ethyl beta-carboline-3-carboxylate	241.10	58.86	1.33	1.51E-04	2.42	P

VIP*: the variable importance in projection value obtained by partial least squares discrimination analysis. Ion mode**: 'P' is positive ion mode, 'N' is negative ion mode.

Table S2. The differential metabolites of *L. reuteri* in CPO were determined by UPLC-Q-TOF-MS/MS, based on VIP > 1, $P < 0.05$.

Compound	mzmed	rtmed	VIP*	P_VALUE	FOLD_CHANGE	Ion mode**
D-Aspartic acid	132.03	451.59	1.62	3.37E-08	0.52	N
D-Ornithine	131.08	561.57	1.60	6.38E-07	0.56	N
3-Hydroxydodecanoic acid	197.16	66.65	1.36	1.85E-03	0.68	N
L-Arginine	173.10	425.24	1.51	1.22E-04	0.69	N
Acetyl-DL-Leucine	172.10	250.00	1.62	2.90E-08	0.69	N
L-Carnosine	225.11	491.04	1.42	1.42E-03	0.70	N
Anthranilic acid	136.04	142.46	1.47	1.98E-04	0.72	N
L-Glutamate	146.05	468.63	1.51	1.11E-04	0.73	N
(S)-2-aminobutyric acid	102.06	442.68	1.53	1.10E-04	0.75	N
L-Anserine	239.12	513.01	1.48	2.88E-04	0.76	N
Indoleacetic acid	174.06	59.85	1.11	3.39E-02	0.78	N
L-Histidine	154.07	577.82	1.43	9.23E-04	0.78	N
Glycerol 3-phosphate	171.01	452.80	1.24	1.13E-02	0.79	N
L-Valine	116.07	367.92	1.49	1.85E-04	0.80	N
L-Pyroglutamic acid	128.04	377.31	1.18	2.24E-02	0.81	N
L-Threonine	118.05	420.64	1.35	3.59E-03	0.81	N
3-Methyl-2-oxopentanoate	129.04	357.76	1.32	5.12E-03	0.82	N
sn-Glycerol 3-phosphoethanolamine	214.06	467.57	1.42	1.26E-03	0.83	N
L-Methionine	148.05	374.86	1.50	1.21E-04	0.83	N
Homocitrate	187.03	414.64	1.24	8.10E-03	0.84	N
Uridine 5-monophosphate	305.03	381.01	1.10	4.07E-02	0.85	N
L-Phenylalanine	164.08	369.48	1.37	2.01E-03	0.85	N
L-Alanine	88.04	447.40	1.36	2.54E-03	0.85	N
Allantoin	157.04	244.61	1.15	2.54E-02	0.85	N
L-Leucine	130.09	364.08	1.23	1.08E-02	0.88	N
L-Glutamine	145.07	448.80	1.03	4.54E-02	0.90	N
D-Proline	114.06	399.39	1.20	1.27E-02	0.93	N
Creatinine	112.05	236.09	1.41	1.64E-03	0.93	N

3-Hydorxy-3-methylglutaric acid	161.05	396.08	1.07	3.41E-02	0.93	N
D-Ribose	149.05	374.41	1.19	2.41E-02	1.05	N
Alpha-D-Glucose	179.06	123.60	1.15	2.96E-02	1.07	N
N-Formylmethionine	176.04	247.22	1.10	3.56E-02	1.07	N
D-Tagatose	179.06	291.67	1.19	1.70E-02	1.08	N
Hydroxyisocaproic acid	131.07	151.72	1.24	1.38E-02	1.09	N
L-Iditol	163.06	362.67	1.35	3.73E-03	1.10	N
D-Sorbitol	182.08	365.29	1.29	1.04E-02	1.13	N
L-Gulonic gamma-lactone	177.03	78.82	1.36	2.74E-03	1.16	N
D-Mannitol	181.08	405.42	1.31	8.58E-03	1.17	N
2-Methyl-3-hydroxybutyric acid	117.06	219.61	1.27	1.02E-02	1.18	N
3,5-Cyclic guanosine monophosphate	344.05	375.41	1.44	1.21E-03	1.22	N
Xylitol	151.06	304.85	1.39	1.72E-03	1.22	N
Sucrose	341.12	275.96	1.43	1.10E-03	1.42	N
Isocitrate	173.01	411.68	1.62	3.99E-08	1.62	N
Citraconic acid	129.02	470.54	1.56	1.48E-05	2.13	N
cis-Aconitate	173.01	467.54	1.61	1.95E-07	2.37	N
alpha-D-Glucose 1-phosphate	259.03	529.44	1.62	8.21E-08	3.11	N
Pro-Glu	245.11	465.00	1.45	2.14E-07	0.35	P
Pro-Ala	187.11	406.38	1.47	5.95E-11	0.41	P
N5-(1-Imino-3-butenyl)-L-ornithine	263.14	296.80	1.45	4.51E-07	0.44	P
N5-(1-Iminoethyl)-L-ornithine	215.14	353.45	1.45	8.00E-08	0.45	P
Pro-Ser	203.10	421.97	1.45	7.81E-08	0.46	P
L-Citrulline	217.12	395.52	1.46	7.28E-08	0.49	P
Antipain	604.35	189.79	1.45	1.69E-07	0.54	P
Heptanoic acid, ethyl ester	355.22	168.03	1.41	1.06E-05	0.54	P
Ornithine	133.10	586.56	1.44	7.22E-07	0.54	P
Pro-Ile	229.15	335.86	1.39	4.10E-05	0.59	P
Pro-Thr	263.11	421.89	1.44	1.40E-06	0.60	P
Val-Tyr-Arg	437.23	138.22	1.40	2.12E-05	0.61	P
Gly-His	213.10	441.76	1.44	4.38E-07	0.61	P
Pro-Gln	244.13	413.11	1.46	1.08E-09	0.62	P
Ser-Ser-Arg	349.18	108.52	1.24	2.36E-03	0.67	P
Leu-His	269.16	356.97	1.23	2.53E-03	0.71	P
PyroGlu-Lys-Arg	414.23	511.06	1.12	1.05E-02	0.73	P
Cellobiose	360.15	465.55	1.31	5.99E-04	0.73	P
Lyso-PC(16:0)	496.34	250.10	1.32	4.80E-04	0.73	P
PyroGlu-Gly-Lys	315.16	529.76	1.12	1.05E-02	0.73	P
Pro-Arg-Arg	428.25	448.49	1.36	1.42E-04	0.74	P
Gln-Met-Arg	434.22	465.81	1.31	5.81E-04	0.75	P
N-(omega)-Hydroxyarginine	173.09	416.33	1.35	1.75E-04	0.77	P
His-Lys	266.16	603.16	1.37	7.45E-05	0.77	P
Histamine	112.09	633.22	1.33	2.78E-04	0.80	P
Pro-His	253.13	439.23	1.13	9.26E-03	0.81	P
Tetraethylene glycol	195.11	76.74	1.07	1.83E-02	0.82	P
4-Aminobutyric acid	104.07	443.02	1.43	1.49E-06	0.83	P
Trp-Thr-Arg	462.24	514.15	1.34	2.62E-04	0.84	P
1-amino-Cyclopropanecarboxylic acid	84.04	448.04	1.31	4.91E-04	0.85	P
Ser-Pro-Lys	372.22	523.97	1.20	4.17E-03	0.85	P
(R)-3-Hydroxybutyric acid	87.04	448.47	1.28	1.00E-03	0.85	P
Pro-Gln-Arg	400.23	565.78	1.10	1.30E-02	0.86	P
Ala-His-Arg	383.20	463.36	1.23	2.64E-03	0.87	P

Dopamine	136.08	405.26	1.32	4.19E-04	0.87	P
Dimethyl sulfone	226.99	479.66	1.24	2.14E-03	0.88	P
L-Tyrosine	182.08	417.65	1.18	5.03E-03	0.89	P
L-Aspartyl-L-phenylalanine	281.11	393.24	1.10	1.35E-02	0.89	P
PyroGlu-Trp-Lys	444.22	519.73	1.13	9.45E-03	0.92	P
Diethanolamine	70.06	380.52	1.03	2.42E-02	1.04	P
PyroGlu-Val-Arg	385.21	422.67	1.13	1.02E-02	1.05	P
Adenosine 3,5-cyclic phosphate	362.08	342.84	1.08	1.63E-02	1.06	P
6,7-Dimethyltetrahydropteri	242.11	385.20	1.07	1.64E-02	1.06	P
N-[(1,1-dimethylethoxy)carbonyl]-L-Proline	279.13	339.25	1.12	1.05E-02	1.07	P
Gln-Phe-Arg	450.23	369.11	1.21	3.69E-03	1.07	P
2-[2-(2-butoxyethoxy)ethoxy]-Ethanol	207.16	9.63	1.16	6.91E-03	1.09	P
Imatinib	494.26	338.38	1.23	2.77E-03	1.09	P
L-N.gamma.-Monomethylarginine	227.10	382.45	1.18	5.24E-03	1.09	P
Asn-Phe-Arg	436.21	384.40	1.03	2.44E-02	1.09	P
Gln-Tyr-Arg	466.20	397.47	1.16	6.90E-03	1.10	P
Phe-Ser-Arg	409.21	357.90	1.06	1.95E-02	1.10	P
PyroGlu-Val-Lys	357.21	418.06	1.07	1.81E-02	1.11	P
N.alpha.-Acetyl-L-lysine	230.15	524.41	1.02	2.82E-02	1.11	P
Pro-Cys-Lys	346.18	316.80	1.02	2.68E-02	1.11	P
1,2-Benzenedicarboxylic acid	149.02	649.10	1.09	1.44E-02	1.11	P
His-Val	237.12	246.73	1.05	2.28E-02	1.14	P
N6-Acetyl-L-lysine	189.12	415.67	1.12	1.09E-02	1.14	P
Ile-Glu	243.13	282.67	1.15	7.50E-03	1.15	P
Cytidine 2,3-cyclic phosphate	306.05	375.75	1.20	4.04E-03	1.15	P
L-Lysine	129.10	313.46	1.02	2.37E-02	1.15	P
Pro-Trp	302.15	309.50	1.02	2.66E-02	1.16	P
PyroGlu-Phe-Arg	433.20	106.36	1.03	2.24E-02	1.16	P
PyroGlu-Met	261.09	313.49	1.22	3.16E-03	1.17	P
Trp-Trp-Arg	547.29	379.20	1.01	2.94E-02	1.17	P
Cys-Ala-Arg	349.16	589.54	1.33	3.01E-04	1.18	P
Choline	104.11	359.99	1.41	1.30E-05	1.18	P
Pro-Asn	230.11	389.82	1.25	1.74E-03	1.19	P
His-Ala	209.10	270.46	1.25	1.97E-03	1.21	P
His-Met	269.10	201.39	1.21	3.79E-03	1.22	P
Stachyose	684.25	538.84	1.11	1.20E-02	1.23	P
His-Ile	251.15	205.34	1.26	1.48E-03	1.24	P
DL-2-Methylglutamic acid	144.07	250.34	1.41	1.18E-05	1.25	P
Trp-Gln	315.14	211.58	1.11	1.29E-02	1.25	P
O-Acetyl-L-serine	148.06	365.17	1.32	3.82E-04	1.26	P
Phe-Trp	334.14	294.58	1.22	2.94E-03	1.26	P
2-Hydroxyadenine	152.06	301.87	1.15	6.94E-03	1.32	P
PyroGlu-His-Arg	423.20	300.60	1.23	2.39E-03	1.35	P
2-hydroxy-Benzeneethanol	121.06	299.04	1.16	6.56E-03	1.36	P
Phe-Ser	235.11	120.00	1.22	2.97E-03	1.37	P
gamma-L-Glutamyl-L-valine	229.12	328.05	1.35	1.64E-04	1.40	P
N-acetyl-L-Methionine	192.07	249.48	1.21	3.29E-03	1.47	P
Quinine	325.19	255.05	1.45	2.23E-07	1.58	P
Glycylproline	214.12	241.69	1.35	1.60E-04	1.60	P
Methylguanidine	74.06	159.77	1.43	2.15E-06	1.61	P
Leu-Gln	242.15	166.90	1.19	4.05E-03	1.62	P
Gly-Glu	204.09	149.58	1.31	5.60E-04	1.64	P

His-Pro	235.12	194.95	1.36	1.01E-04	1.70	P
Pyridoxine	170.08	127.54	1.25	1.81E-03	1.78	P
2-O-methyladenosine	282.12	166.06	1.32	3.90E-04	2.03	P
3-Pyridinemethanol	110.06	87.17	1.41	9.02E-06	2.04	P
5-Methylcytosine	126.07	251.86	1.45	3.56E-07	2.05	P
Tyr-Pro	261.12	132.27	1.47	3.08E-10	2.25	P
N,N-dimethyl-1H-Purin-6-amine	164.09	93.94	1.34	2.51E-04	2.25	P
Benzeneethanamine	185.11	242.33	1.46	1.53E-08	2.28	P
Tryptamine	224.12	243.75	1.28	1.04E-03	2.33	P
Ile-Leu	227.17	62.59	1.36	1.25E-04	2.74	P
Maltotriose	522.20	523.26	1.45	1.90E-07	2.85	P
Ethyl beta-carboline-3-carboxylate	241.10	58.86	1.43	1.88E-06	2.99	P
Adenosine 2,3-cyclic monophosphate	330.06	327.31	1.46	3.36E-08	3.04	P
Phe-Pro	245.13	67.59	1.46	4.77E-09	4.75	P

VIP*: the variable importance in projection value obtained by partial least squares discrimination analysis. Ion mode**: 'P' is positive ion mode, 'N' is negative ion mode.

Table S3. The differential metabolites of *L. reuteri* in CPR were determined by UPLC-Q-TOF-MS/MS, based on VIP > 1, *P* < 0.05.

Compound	mzmed	rtmed	VIP*	P_VALUE	FOLD_CHANGE	Ion mode**
3-Hydroxydodecanoic acid	197.16	66.65	1.59	1.00E-06	0.17	N
Uracil	111.02	138.31	1.52	4.37E-05	0.43	N
D-Aspartic acid	132.03	451.59	1.61	3.24E-08	0.53	N
O-Acetyl-L-serine	163.06	229.00	1.54	1.99E-05	0.55	N
Pentadecanoic Acid	241.22	63.18	1.54	2.89E-05	0.60	N
L-Arginine	173.10	425.24	1.35	2.36E-03	0.74	N
D-Ornithine	131.08	561.57	1.47	2.67E-04	0.84	N
3-Methyl-2-oxopentanoate	129.04	357.76	1.08	3.19E-02	0.91	N
Acetylglycine	116.04	354.37	1.15	1.63E-02	0.91	N
L-Methionine	148.05	374.86	1.14	2.41E-02	0.93	N
Acetyl-DL-Leucine	172.10	250.00	1.06	4.39E-02	0.94	N
Salicylic acid	137.03	59.04	1.04	4.58E-02	0.94	N
Creatinine	112.05	236.09	1.13	3.08E-02	1.06	N
Ribitol	133.05	276.10	1.08	3.33E-02	1.06	N
alpha-ketoglutarate	145.02	410.19	1.17	2.12E-02	1.06	N
2-Methyl-3-hydroxybutyric acid	117.06	219.61	1.09	4.10E-02	1.06	N
alpha-N-Acetyl-L-glutamine	187.08	365.32	1.17	1.43E-02	1.07	N
Citrate	191.02	643.62	1.36	2.27E-03	1.10	N
Adenine	134.05	278.14	1.22	1.45E-02	1.12	N
N-Acetyl-L-alanine	130.05	309.95	1.31	4.99E-03	1.12	N
DL-3-Phenyllactic acid	165.06	120.87	1.30	3.87E-03	1.12	N
Alpha-D-Glucose	179.06	123.60	1.43	6.60E-04	1.12	N
Isocitrate	173.01	411.68	1.10	2.82E-02	1.13	N
D-Mannose	201.03	408.91	1.25	9.57E-03	1.13	N
3-Hydroxyisovaleric acid	155.00	539.09	1.45	4.78E-04	1.13	N
Myristic acid	227.21	664.72	1.49	1.28E-04	1.14	N
L-Threonate	135.03	370.46	1.39	1.86E-03	1.14	N
N6-Acetyl-L-lysine	187.11	415.61	1.48	1.51E-04	1.15	N
Adenosine 3-monophosphate	346.07	464.06	1.39	2.16E-03	1.15	N
2-Oxoadipic acid	141.02	549.18	1.31	4.97E-03	1.16	N
Glycerophosphocholine	256.10	452.48	1.05	4.17E-02	1.16	N
Formylanthranilic acid	164.04	63.10	1.24	1.14E-02	1.17	N

Hydroxyisocaproic acid	131.07	151.72	1.49	1.82E-04	1.17	N
DL-lactate	89.03	237.36	1.40	1.53E-03	1.17	N
Indolelactic acid	204.07	197.72	1.33	3.18E-03	1.18	N
N-Formylmethionine	176.04	247.22	1.29	7.45E-03	1.19	N
Taurochenodeoxycholate	498.27	301.18	1.25	1.19E-02	1.19	N
Heptadecanoic acid	269.26	660.71	1.32	3.94E-03	1.19	N
alpha-D-Glucose 1-phosphate	259.03	529.44	1.19	1.60E-02	1.20	N
L-Histidine	154.07	577.82	1.44	7.75E-04	1.21	N
Hydroxyphenyllactic acid	181.06	256.68	1.42	6.73E-04	1.22	N
D-gluconate	195.06	400.12	1.46	2.57E-04	1.23	N
cis-Aconitate	173.01	467.54	1.25	7.65E-03	1.27	N
Citraconic acid	129.02	470.54	1.11	3.00E-02	1.29	N
(S)-2-Hydroxyglutarate	147.03	440.62	1.43	7.27E-04	1.31	N
Trehalose	341.12	430.20	1.40	1.49E-03	1.33	N
4-Pyridoxic acid	182.05	55.08	1.20	1.36E-02	1.38	N
Xylitol	151.06	304.85	1.49	1.59E-04	1.39	N
L-Gulonic gamma-lactone	177.03	78.82	1.58	1.87E-06	1.42	N
Azelaic acid	187.10	386.06	1.40	7.85E-04	1.51	N
Gly-His	213.10	441.76	1.72	2.08E-05	0.71	P
Histamine	112.09	633.22	1.45	5.23E-03	0.75	P
Heptanoic acid, ethyl ester	355.22	168.03	1.42	6.95E-03	0.76	P
Pro-Glu	245.11	465.00	1.61	4.74E-04	0.77	P
Ornithine	133.10	586.56	1.65	2.69E-04	0.78	P
Lyso-PC(16:0)	496.34	250.10	1.66	2.07E-04	0.79	P
L-Carnosine	227.11	491.80	1.70	1.04E-04	0.80	P
6,7-Dimethyltetrahydropterin	242.11	385.20	1.70	6.91E-05	0.81	P
Norfloxacin	302.13	430.90	1.59	8.91E-04	0.81	P
Val-Tyr-Arg	437.23	138.22	1.59	7.01E-04	0.81	P
Antipain	604.35	189.79	1.65	1.94E-04	0.83	P
Pro-Asn	230.11	389.82	1.55	1.19E-03	0.84	P
Stachyose	684.25	538.84	1.27	2.21E-02	0.84	P
L-Anserine	241.13	514.75	1.63	4.42E-04	0.85	P
Glu-Asp-Lys	373.17	476.68	1.18	3.72E-02	0.85	P
Gln-Met-Arg	434.22	465.81	1.36	1.08E-02	0.87	P
3,5-Cyclic guanosine monophosphate	346.05	383.21	1.56	1.75E-03	0.88	P
L-Aspartyl-L-phenylalanine	281.11	393.24	1.16	4.30E-02	0.89	P
L-Pipecolic acid	147.11	646.08	1.36	1.22E-02	0.89	P
Pro-Ala	187.11	406.38	1.68	8.51E-05	0.89	P
Trp-Thr-Arg	462.24	514.15	1.35	1.32E-02	0.89	P
Pro-Arg-Arg	428.25	448.49	1.25	2.68E-02	0.90	P
Ala-His-Arg	383.20	463.36	1.33	1.33E-02	0.91	P
L-Threonine	120.07	446.25	1.15	4.72E-02	0.91	P
Gln-His-Arg	440.21	467.42	1.23	2.71E-02	0.92	P
4-Aminobutyric acid	104.07	443.02	1.45	4.63E-03	0.93	P
1-amino-Cyclopropanecarboxylic acid	84.04	448.04	1.41	6.77E-03	0.93	P
Pro-Gln	244.13	413.11	1.25	2.73E-02	0.94	P
Asn-Phe-Arg	436.21	384.40	1.31	2.17E-02	1.06	P
Gln-Phe-Arg	450.23	369.11	1.41	1.07E-02	1.06	P
Tyramine	120.08	325.26	1.30	2.65E-02	1.06	P
PyroGlu-Val-Lys	357.21	418.06	1.45	4.98E-03	1.07	P
His-Ile	251.15	205.34	1.44	7.10E-03	1.08	P
Agomelatine	244.14	557.20	1.42	7.62E-03	1.08	P

1,2-Benzenedicarboxylic acid	149.02	649.10	1.34	1.91E-02	1.08	P
Pro-Trp	302.15	309.50	1.17	4.93E-02	1.08	P
gamma-L-Glutamyl-L-valine	229.12	328.05	1.33	1.34E-02	1.08	P
Imatinib	494.26	338.38	1.43	7.96E-03	1.08	P
Gln-Tyr-Arg	466.20	397.47	1.40	1.24E-02	1.09	P
PyroGlu-Tyr-Arg	449.20	335.22	1.46	4.35E-03	1.09	P
Pro-Val	278.16	534.70	1.30	2.29E-02	1.10	P
Phe-Ser-Arg	409.21	357.90	1.39	1.34E-02	1.12	P
N.alpha.-Acetyl-L-lysine	230.15	524.41	1.64	5.50E-04	1.12	P
Choline	104.11	359.99	1.73	4.08E-05	1.15	P
Dimethyl sulfone	226.99	479.66	1.58	1.70E-03	1.17	P
Trp-Gln	315.14	211.58	1.15	4.14E-02	1.18	P
Leu-Gln	242.15	166.90	1.43	6.74E-03	1.19	P
Pro-Cys-Lys	346.18	316.80	1.64	3.84E-04	1.20	P
His-Pro	235.12	194.95	1.49	4.30E-03	1.20	P
DL-O-tyrosine	245.09	148.35	1.32	1.81E-02	1.20	P
His-Ala	209.10	270.46	1.62	4.99E-04	1.20	P
2-O-methyladenosine	282.12	166.06	1.19	4.05E-02	1.21	P
Phe-Ser	235.11	120.00	1.64	4.66E-04	1.26	P
N,N-dimethyl-1H-Purin-6-amine	164.09	93.94	1.45	4.77E-03	1.44	P
5-Methylcytosine	126.07	251.86	1.43	6.22E-03	1.58	P
Tyr-Pro	261.12	132.27	1.57	1.13E-03	1.62	P
Ile-Leu	227.17	62.59	1.34	1.53E-02	1.69	P
Phe-Pro	245.13	67.59	1.50	3.24E-03	1.81	P
Ethyl beta-carboline-3-carboxylate	241.10	58.86	1.26	2.75E-02	1.88	P
Sucrose	360.14	429.54	1.81	8.33E-11	2.80	P

VIP*: the variable importance in projection value obtained by partial least squares discrimination analysis. Ion mode**: ‘P’ is positive ion mode, ‘N’ is negative ion mode.

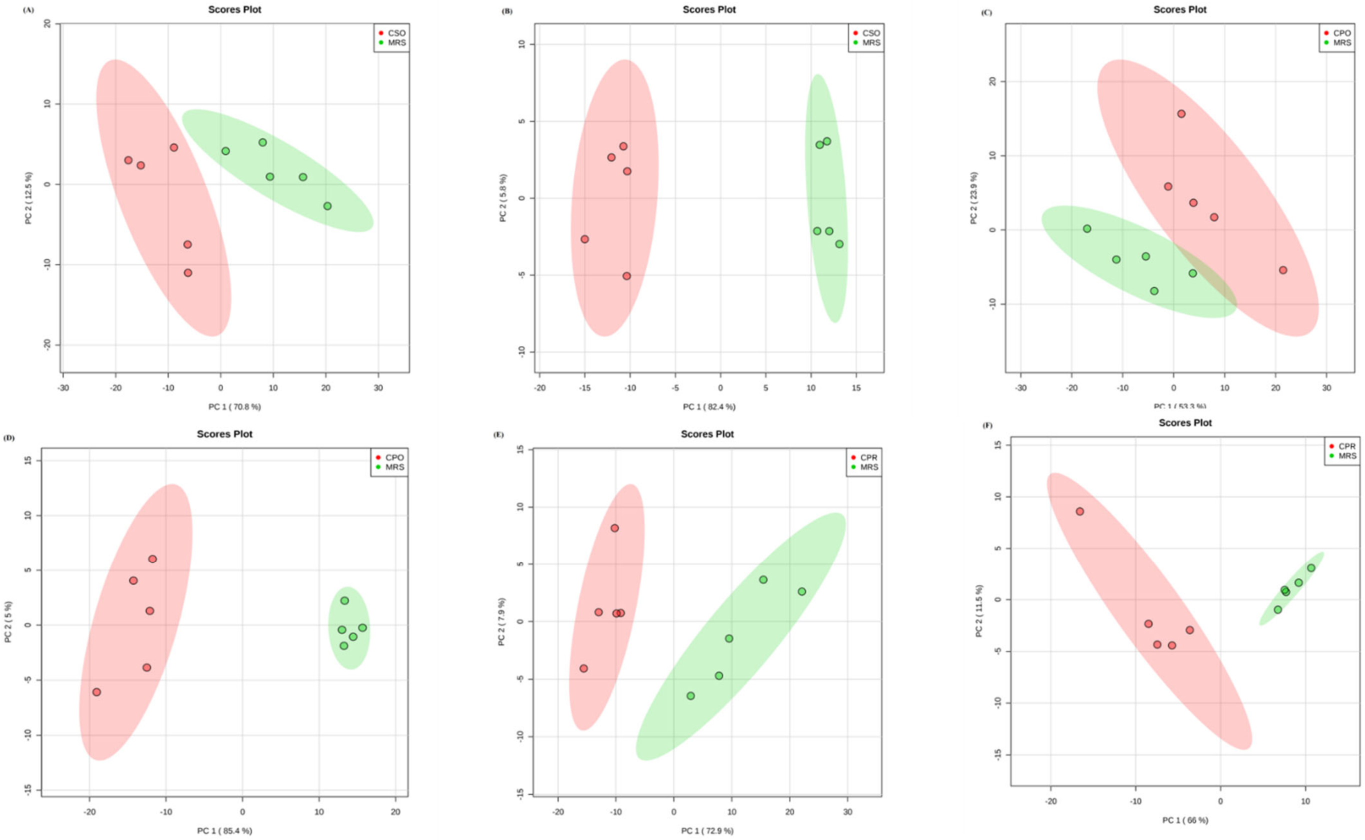

Figure S1. Principal component analysis of differential metabolites in different extracts of *L. reuteri* under positive ion mode and negative ion mode. Positive ion mode: A (CSO vs. MRS), C (CPO vs. MRS), E (CPR vs. MRS). Negative ion mode: B (CSO vs. MRS), D (CPO vs. MRS), F (CPR vs. MRS).

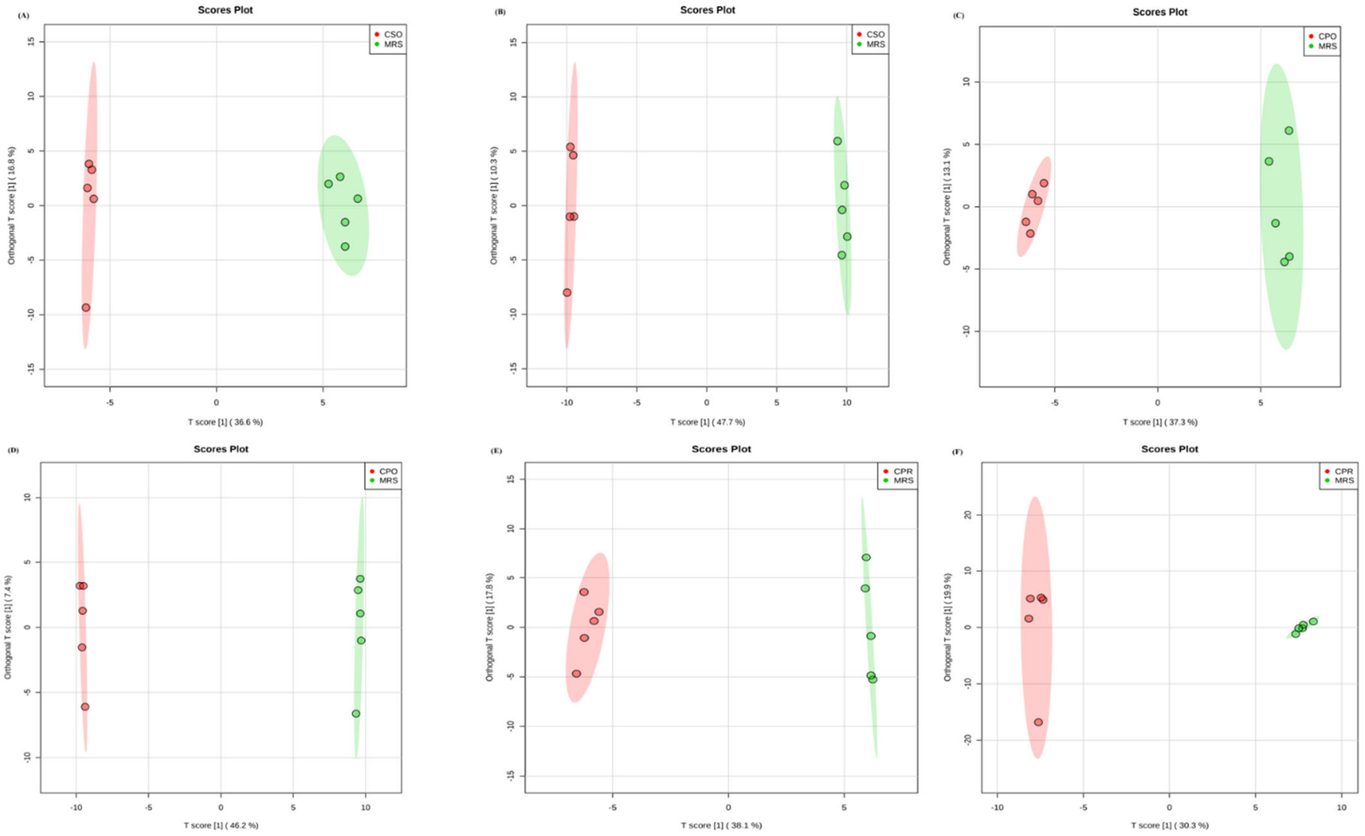

Figure S2. Orthogonal partial least square discriminant analysis of differential metabolites in different extracts of *L. reuteri*

under positive ion mode and negative ion mode. Positive ion mode: A (CSO vs. MRS), C (CPO vs. MRS), E (CPR vs. MRS). Negative ion mode: B (CSO vs. MRS), D (CPO vs. MRS), F (CPR vs. MRS).
